# B‐cells with a FasL expressing regulatory phenotype are induced following successful anti‐tuberculosis treatment

**DOI:** 10.1002/iid3.140

**Published:** 2016-12-27

**Authors:** Ilana C. van Rensburg, Léanie Kleynhans, Alana Keyser, Gerhard Walzl, Andre G. Loxton

**Affiliations:** ^1^ Division of Molecular Biology and Human GeneticsFaculty of Medicine and Health SciencesSA MRC Centre for TB ResearchDST/NRF Centre of Excellence for Biomedical Tuberculosis ResearchStellenbosch UniversityCape TownSouth Africa; ^2^ Clinical Laboratory SciencesFaculty of Health SciencesUniversity of Cape TownCape TownSouth Africa

**Keywords:** Apoptosis, broncho‐alveolar lavage fluid, FasL, immune exhaustion

## Abstract

**Introduction:**

Studies show that B‐cells, in addition to producing antibodies and antigen‐presentation, are able to produce cytokines as well. These include regulatory cytokines such as IL‐10 by regulatory B‐cells. Furthermore, a rare regulatory subset of B‐cells have the potential to express FasL, which is a death‐inducing ligand. This subset of B‐cells have a positive role during autoimmune disease, but has not yet been studied during tuberculosis. These FasL‐expressing B‐cells are induced by bacterial LPS and CpG, thus we hypothesized that this phenotype might be induced during tuberculosis as well.

**Methods:**

B‐cells from participants with TB (at diagnosis and during treatment) and controls were collected, and analyzed by means of real‐time PCR and flow cytometry. In addition to this, BAL was collected from TB participants as well and analyzed by means of MAGPix (multi‐cytokine) technology.

**Results:**

Gene expression analysis show that FASL transcript levels increase by the end of treatment. Similarly, phenotypic analysis show that there is a higher frequency of FasL‐expressing B‐cells by the end of treatment.

**Conclusion:**

Collectively, these results indicate that these FasL‐expressing B‐cells are being induced during anti‐TB treatment, and thus may play a positive role. Further studies are required to elucidate this.

## Introduction

B lymphocytes (B‐cells) are predominantly recognized for their role during humoral immunity, whereby these cells produce antibodies to assist in fighting infections. However, B‐cells are able to produce cytokine as well, and the type of cytokines produced is dependent on the microenvironment 1. Effector type 1 B‐cells (Be‐1) produce Th1 type cytokines such as interferon gamma (IFN‐γ) and tumor necrosis factor alpha (TNF‐α), Be‐2 cells produce Th2 type cytokines while regulatory B‐cells produce IL‐10 2. Cytokine‐producing B‐cells play important roles in the regulation of immunity and have been extensively studied in the context of autoimmune disease 2, 3. B‐cell culture studies have shown that there is a balance in the proinflammatory and regulatory responses of B‐cells 4, 5, 6. In the context of autoimmune disease, and more specifically multiple sclerosis (MS), this balance is skewed toward a more proinflammatory response due to a reduction in the production of IL‐10 3. The balance is however restored upon treatment. An additional regulatory function utilized by B‐cells is the expression of Fas‐Ligand (FasL) upon activation 7, 8. This subset of B‐cells is rare and has been studied in the context of autoimmune disease as well as helminth infections 9. These regulatory “killer” B‐cells are CD19^+^IgM^+^ and induce apoptosis of CD4 T‐cells, and thus have a positive effect during autoimmune disease. The B‐cells induce FasL‐mediated apoptosis of autoreactive T‐cells, thereby regulating/reducing the inflammation. Klinker and coworkers (2013) showed that this regulatory subset of B‐cells are induced by interleukin(IL)‐5, and that there is an increase in the expression of IL‐5 receptor α (IL‐5Rα) 9. Lymphocytes may undergo exhaustion due to persistent infections and prolonged activation. This occurs as the cells fail to respond to antigenic challenge, and has increased expression of markers of exhaustion such as programmed cell death protein (PD‐1). The role of these “killer” B‐cells during tuberculosis has not yet been studied, but it was shown that this phenotype is induced in B‐cells upon stimulation with TLR9 agonist CpG 10. This suggests that the same regulatory phenotype could be induced in the context of TB, as B‐cell expression of TLR9 is triggered by mycobacterial CpG motifs 11, 12, 13, 14. Thus, we hypothesized that FasL expression on B‐cells is induced during *Mycobacterium tuberculosis* infection. We therefore aimed to evaluate gene expression patterns of FASL and IL5RA by B‐cells using real‐time PCR. Additionally, we aimed to evaluate the phenotype of B‐cells from individuals with TB by means of flow cytometry, and how this changes throughout treatment. Finally, we evaluated cytotoxic host marker levels in BAL and plasma from individuals with TB.

## Materials and Methods

### Ethics statement

Ethical approval was obtained from the ethics committee of Stellenbosch University (N10/01/013 and N13/05/064) and the City of Cape Town City Health. The study was conducted according to the Helsinki Declaration and International Conference of Harmonization guidelines. Written informed consent was obtained from all study participants.

### Participant recruitment

All participants were recruited in the Ravensmead/Uitsig Community of Cape Town, South Africa. Participants newly diagnosed and who had a first episode of TB were recruited for the study before initiation of treatment. The inclusion criteria is shown in Table 1. The TB cases were followed over the course of 6 month treatment. In addition, healthy controls (CTRL) from the same community were also recruited. The healthy controls were all QuantiFERON (QFN) positive, which is indicative of latent TB infection (LTBI). Participants with cancers were included in the study as controls for the bronchoscopies performed on TB cases. These participants displayed respiratory symptoms due to inflammatory or infective cause, but were *M. tuberculosis* sputum‐culture and GeneXpert negative, which indicated that they did not have active TB disease. Individuals who had recently completed anti‐TB treatment (within 3 months), were *M. tuberculosis* sputum‐culture negative and who consented were selected for bronchoscopies. Bronchoscopies were performed by an experienced pulmonologist at Tygerberg Hospital, Cape Town, and transported to the laboratory under controlled conditions for processing.

**Table 1:** Inclusion criteria for active TB cases and control (CTRL) participant recruitment

TB	CTRL
Clinical signs of TB^1^ Chest radiographs with sign of TB Positive sputum culture test^2^ HIV negative	No clinical signs of TB^1^ Chest radiographs with no signs of TB Negative sputum culture test QFN positive HIV negative

^1^Cough, fever, night sweats, weight loss, loss of appetite.

^2^Culture negative TB included (if GeneXpert positive).

### B‐cell phenotype analysis by flow cytometry

A 1 ml of sodium heparin blood was collected from 15 CTRLs (once‐off) and 13 TB cases at diagnosis, week 2, months 1 and 6 of treatment (Supplementary Table S1). The blood was analysed using 1× BD FACS Lysing solution (BD), and the leukocytes were cryopreserved in 10% DMSO, Saarchem), placed in a Mr. Frosty at −80°C overnight and transferred to liquid nitrogen the following day.

Once all the samples were received, cells were retrieved from liquid nitrogen and washed in FACS buffer (PBS, Biowhittaker, MD, USA) containing 2% FCS). The cells were then stained for 30 min with the following antibodies; CD19‐BV510, IgM‐FITC, CD125w (or IL5RA)‐PE, CD3‐PerCP, PD1‐BV421, CD40‐APC‐H7, CD38‐PE‐Cy7, and CD178 (or FASL)‐APC. Subsequently, the samples were acquired on a BD FACS Canto II and the data analyzed using FlowJo v10 software (Oregon, USA). To determine the appropriate gating cut‐off, to increase the accuracy of distinguishing different populations, Fluorescence‐minus‐one (FMO) control samples were utilized 15.

### B‐cell gene expression analysis

Blood was collected into sodium heparin tubes from 19 TB cases at diagnosis, months 2 and 6 of treatment (Supplementary Table S2), and peripheral blood mononuclear cells (PBMCs) were isolated using the ficol‐histopaque (Sigma–Aldrich, Missouri, USA) separation method. Subsequently, total B‐cells were isolated from the PBMCs by positive selection using the B‐cell MACS CD19 Microbeads, (Miltenyi) according to the manufacturer's instructions. Cell purities were checked by flow cytometry and were above 90%.

RNA was isolated from these B‐cells and used to synthesize cDNA. The procedure was carried out in a thermal cycler (Life Technologies, California, USA) using the First Strand Kit (Hilden, Germany) according to the manufacturer's instructions. Quantitative PCR was carried out using the ABI 7900HT platform (Applied Biosystems, California, USA). The RT^2^ Profiler Custom Arrays (Qiagen) were utilized and manufacturer's instructions were followed. The arrays contained primers for the following genes: Fas‐ligand (*FASLG*) (NM000639.2) and IL5RA receptor alpha (*IL5RA*) (NM_000564.4) as well as two housekeeping genes *B2M* (NM_004048) and *GAPDH* (NM_002046). *FASLG and IL5RA* is associated with regulatory B‐cells.

The gene expression data obtained from the ABI 7900HT was represented as Ct values, which indicated the earliest visible cycle of amplification. These Ct values were converted to fold change values using the Qiagen online software (www.SABiosciences.com/pcrarraydataanalysis.php).

### Host marker detection in BAL fluid and PLASMA using MAGPix platform

Bronchoscopies were performed on a group of TB patients at diagnosis and another group of TB patients at the end of treatment (Supplementary Table S3). During the procedure, broncho‐alveolar lavage (BAL) was collected by washing the lung interior with saline. The BAL samples were spun down and the supernatants were used for protein analysis. In addition, blood was collected from these individuals into EDTA tubes and centrifuged in order to collect plasma. Bronchoscopies were also performed on 10 individuals with lung cancer, to serve as (other lung‐disease) controls, for the collection of BAL samples. A custom Human CD8+ T‐cell Magnetic Bead Panel Kit (HCD8MAG15K17PMX, Merck Millipore, St. Charles, MO) was utilized to evaluate the levels of the following six host markers: Granzyme A, Granzyme B, Perforin, soluble Fas Ligand (sFasL), Interleukin (IL) 5, and Granulocyte macrophage colony stimulating factor (GM‐CSF). The assay was carried out following the manufacturer's instructions and the concentration of the marker measured on the MAGPix platform (Bio‐Rad Laboratories, California, USA). Two quality controls included in the kit were run in duplicate. Levels of all analytes in the quality controls were within the expected ranges (pg/ul). A standard curve ranging from 0.08 to 1.7 (pg/ul) was used for GM‐CSF, 0.93–19 (pg/ul) for Granzyme A, 0.04–0.85 (pg/ul) for Granzyme B, 0.03–0.67 (pg/ul) for IL‐5, 0.27–6.0 (pg/ul) for sFasL, and 0.24–5.6 (pg/ul) for Perforin. Bio‐Plex Manager software (California, USA), version 6.1, was used to analyze the data.

### Statistical analysis

Statistical analysis was carried out using Graph Pad Prism 5 Software (San Diego, CA). Where applicable, differences between groups were calculated using Student *t*‐test, one‐way ANOVA and Dunn's multiple comparison post‐hoc tests. Significant differences are indicated by *P *< 0.05.

## Results

### Activated B‐cells express a FasL + IL5Ra+ phenotype following anti‐TB treatment

**Figure 1:**
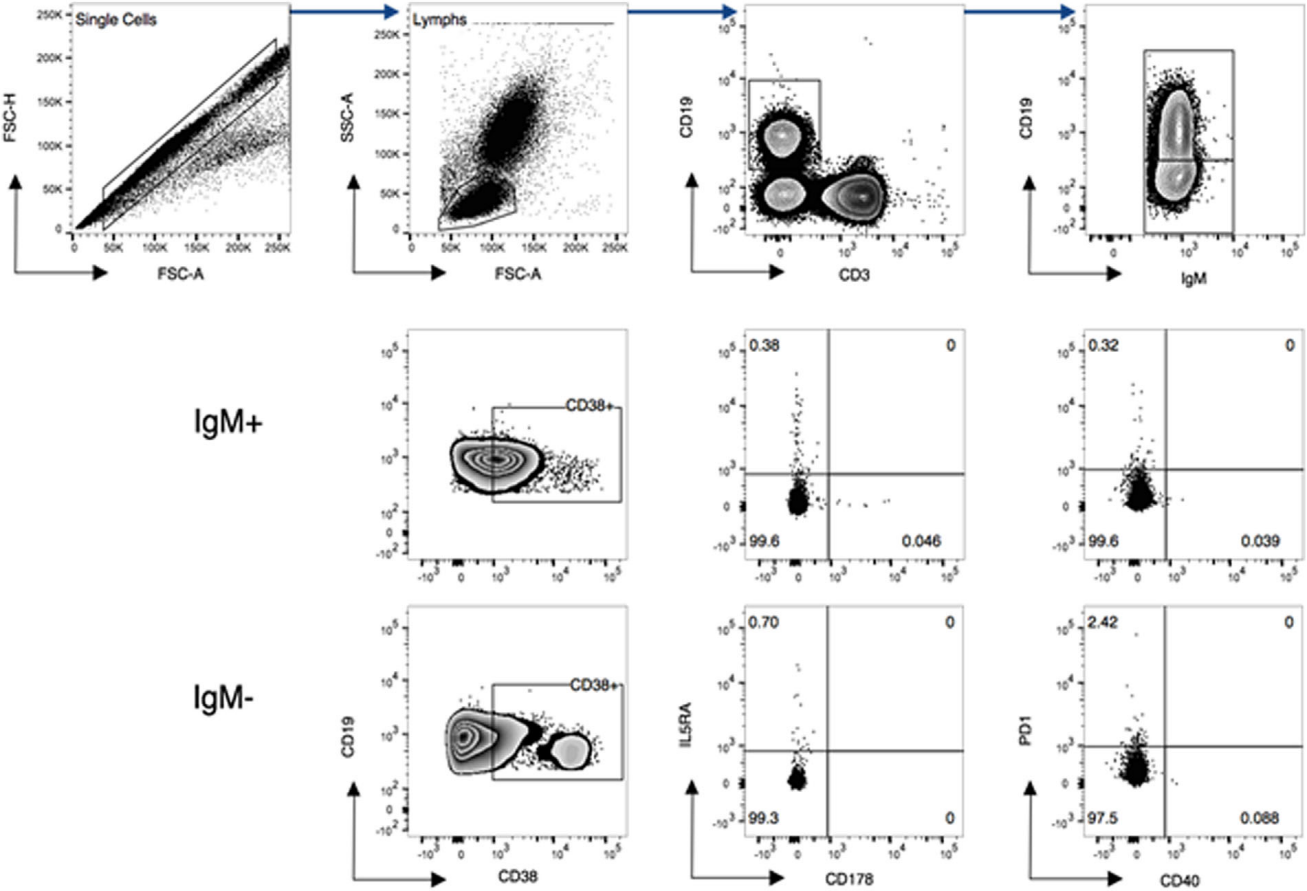
The Gating strategy used to identify B‐cells of interest. Single cells were selected and lymphocytes gated based on the light scatter of the cells. Total lymphocytes were plotted (CD3) and the population of interest, CD19+ cells were gated for IgM^+^ and IgM^−^ subsets, and the expression of CD38, CD178, and CD40 evaluated.

Studies have shown that there is a subset of regulatory B‐cells that express FASL, and that these cells are induced by IL‐5, with an increased expression of IL5RA, thus we were interested in the expression of FASL (CD178) and IL5RA (CD125) on B‐cells. Additionally, we were interested in the expression of PD1 and CD40 as well, as increased expression of these receptors are linked to cell exhaustion. To evaluate the expression of the surface markers, the gating strategy shown in Figure 1 was followed.

**Figure 2:**
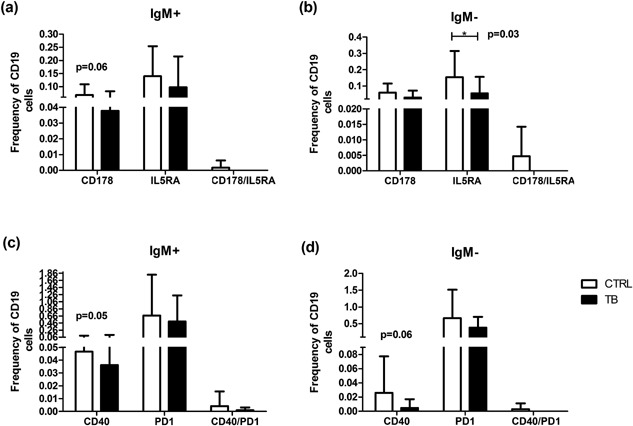
Frequency of IgM^−^ and IgM^+^ B‐cells expressing FASL (CD178)/IL5Ra (CD125) and CD40/PD1 between TB cases and CTRLs. CD19^+^ B‐cells acquired by means of Flow cytometry. Differences in frequency of cells expressing regulatory and exhaustion markers calculated using one‐way ANOVA tests and Dunn's multiple comparison post‐hoc tests. Significant differences are indicated by an asterisk (*), where *P* < 0.05 (a) IgM^+^ B‐cells expressing FASL/CD178 and IL5RA/CD125 (b) IgM^−^ B‐cells expressing FASL/CD178 and IL5RA/CD125 (c) IgM^+^ B‐cells expressing CD40 and PD1 (d) IgM^−^ B‐cells expressing CD40 and PD1. TB cases *n* = 13 and CTRLs *n* = 15.

When comparing the frequency of CD19^+^ IgM^+^CD38^+^ cells expressing FASL (CD178) and IL5RA (CD125) between CTRLs and TB cases, there were no significant differences between the two groups (Fig. 2a). There was, however, a trend that the expression of FASL (CD178, *P* = 0.06), was higher in CTRLs when compared to TB cases. When comparing the frequency CD19^+^ IgM^−^CD38^+^ cells expressing FASL/CD178 and IL5RA/CD125, we found that there was a significant difference in the frequency of IL5RA between CTRL and TB groups (*P* = 0.03; Fig. 2b). There were no significant differences in frequency of CD19^+^ IgM^+^CD38^+^ and CD19^+^ IgM*^−^*CD38^+^ expressing CD40 between CTRLs and TB participants. PD1 (Fig. 2c and d).

There was, also, however a trend in that the expressing of CD40 by IgM^−^ B‐cells, where there was a higher expression in CTRLs (*P* = 0.06). Overall, the expression of FASL/CD178 and IL5RA/CD125, as well as CD40 and PD1 was higher in CTRLs compared to TB participants (Fig. 2), even though these differences were not statistically significant.

### Effects of anti‐TB treatment on the expression of regulatory and exhaustion markers by B‐cells

**Figure 3:**
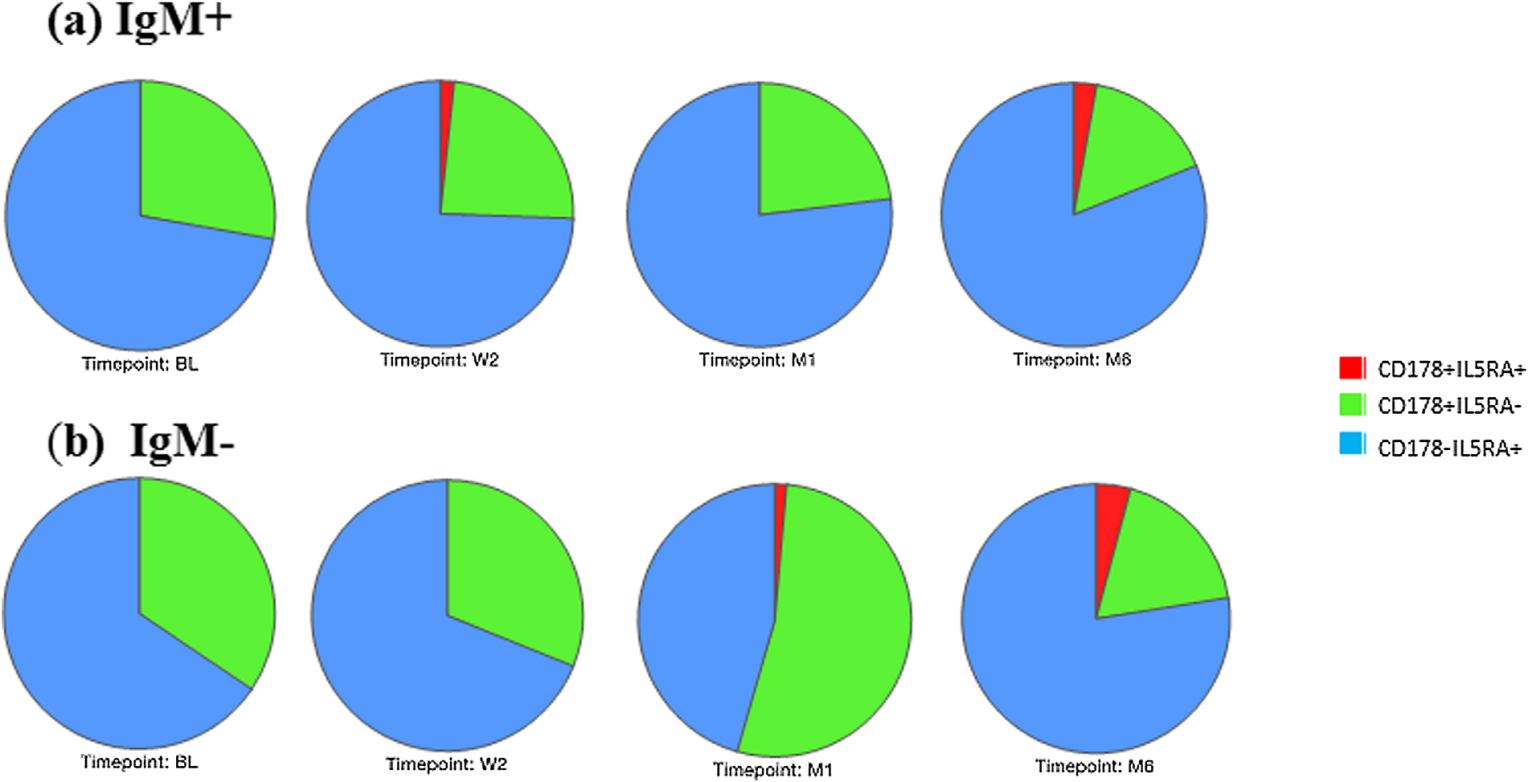
Frequencies of Regulatory B‐cells during treatment. Pie charts depict frequencies of B‐cells expressing either CD178, IL5RA, or both simultaneously in TB participants during treatment. Where BL is diagnosis, W2 is week 2, M1 is month 1, and M6 is month 6 of treatment. (a) Frequencies of IgM positive B‐cells and (b) Frequencies of IgM negative B‐cells. TB cases *n* = 13.

The frequency of CD19^+^IgM^+^CD38^+^ and CD19^+^IgM^−^CD38^+^ cells expressing FASL/CD178 and IL5RA/CD125, as well as CD40 and PD1 were evaluated over the course of 6‐month anti‐TB treatment. Figure 3 shows the changes in the expression of FASL and IL5RA following TB treatment in order to assess the effects of treatment. The frequency of CD19^+^IgM^+^CD38^+^ of cells expressing FASL/CD178 (CD178^+^IL5RA^−^) decreased during treatment, whereas the expression of IL5RA/CD125 (CD178^−^IL5RA^+^) increased during treatment (Fig. 3a). Furthermore, the frequency of cells expressing both FASL/CD178 and IL5RA/CD125 (CD178^+^IL5RA^+^) was higher at week 2 and month 6 of treatment compared to diagnosis (Fig. 3a). These changes were, however, not statistically significant. The frequency of CD19^+^IgM^−^CD38^+^ cells expressing FASL/CD178 decreased by month 6 of treatment, however expression was highest at month 1 of treatment (Fig. 3b). The expression of IL5RA/CD125 increased by the end of treatment, but was lowest at month 1 of treatment. (Fig. 3b). Additionally, the frequency of cells expressing FASL/CD178 and IL5RA/CD125 (CD178^+^IL5RA^+^) was higher at month 1 and month 6 of treatment compared to diagnosis. These changes, however, were not statistically significant either.

**Figure 4:**
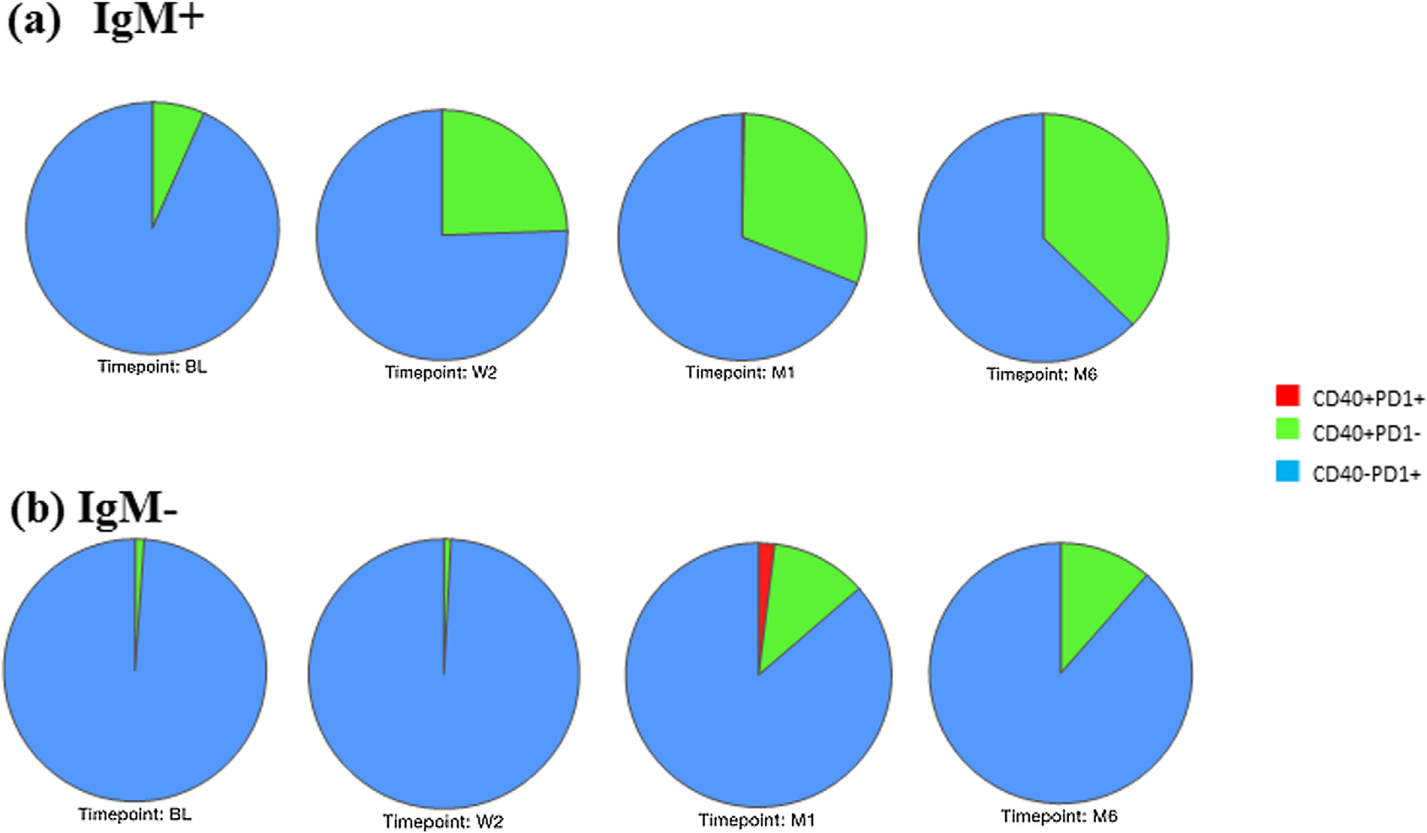
Frequencies of Regulatory B‐cells during treatment. Pie charts depict frequencies of B‐cells expressing either CD40, PD1, or both simultaneously in TB participants during treatment. Where BL is diagnosis, W2 is week 2, M1 is month 1, and M6 is month 6 of treatment. (a) Frequencies of IgM positive B‐cells and (b) Frequencies of IgM negative B‐cells. TB cases *n* = 13.

The expression of CD40 and PD1 by CD19^+^IgM^+^CD38^+^ and CD19^+^IgM^−^CD38^+^ cells were evaluated as well (Fig. 4). The frequency of IgM^+^ and IgM^−^ B‐cells expressing CD40 (CD40^+^PD1^−^) increased during treatment, with the increase being more apparent in the IgM^+^ population, while the frequency of PD1 expressing (CD40^−^PD1^+^) B‐cells decreased (Fig. 4a and b). The frequency of IgM^−^ B‐cells expressing both CD40 and PD1 (CD40^+^PD1^+^) was elevated at month 1; however, none of the latter changes were statistically significant.

### Expression of genes linked to regulatory phenotype during treatment

**Figure 5:**
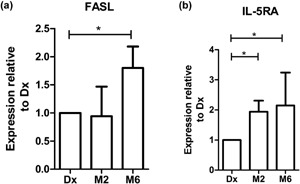
Genes differentially expressed during anti‐TB treatment. Relative expression of genes as calculated using the delta‐delta Ct method. Statistical differences calculated by means of Student *t*‐test, where significant differences are indicated by an asterisk (*P ≤ 0, 05). Data represented as mean fold change with bars representing SEM. Positive values indicate increases in gene expression, and negative values indicate decreases in gene expression relative to diagnosis. TB cases *n* = 19.

RT‐qPCR was used to evaluate the expression of *FASL* and *IL5RA* transcripts in B‐cells from TB participants during treatment (diagnosis, months 2 and 6). When the expression of genes were compared between the 3‐time points, *FASL* expression was found to be significantly upregulated at month 6 compared to diagnosis (*P* = 0.01; Fig. 5a). Similarly, the expression of *IL5RA* was significantly upregulated at month 2 (*P* = 0.01) and month 6 (*P* = 0.01; Fig. 5b) compared to diagnosis.

### Changes in BAL‐secreted cytokines following anti‐TB treatment but not in the plasma of TB cases

**Figure 6:**
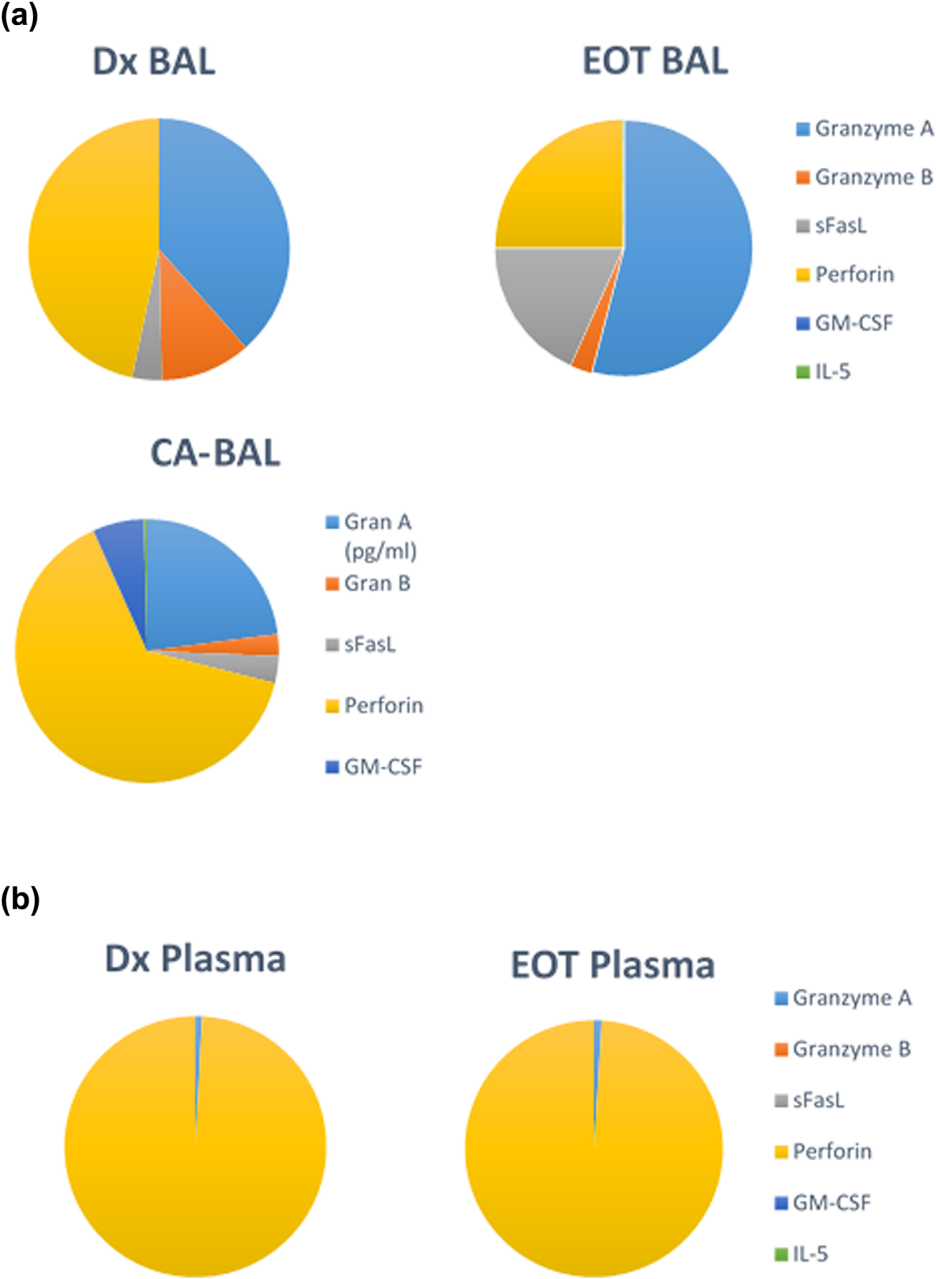
Comparison of analyte levels in BAL fluid and Plasma from TB cases at diagnosis (Dx), end of treatment (EOT), and cancer controls. Diagnosis *n* = 8, end of treatment *n* = 20, and cancer controls (CA) *n* = 10. Levels of the analytes in BAL and plasma are represented as percentages within the respective groups. (a) Analyte composition in BAL from TB cases at diagnosis (Dx) and end of treatment (EOT), and cancer (CA) controls and (b) Analyte composition in Plasma from TB cases at Dx and EOT.

**Figure 7:**
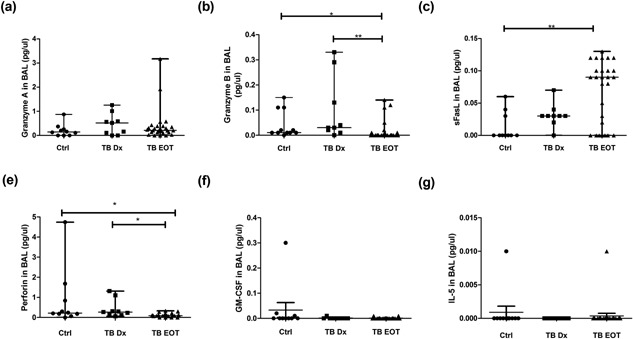
Comparison of analyte levels in BAL fluid between Ctrls, TB participants at diagnosis, and end of treatment (EOT). Analyte levels (pg/ul) were measured using MAGPix technology. ANOVA tests were performed to determine significant differences (*P* ≤ 0.05) between groups, which is indicated with an asterisk (*). (a) Granzyme A levels, (b) Granzyme B levels, (b) sFasL levels, (d) Perforin levels, (e) GM‐CSF levels, and (f) IL‐5 levels in the respective groups. TB cases Dx *n* = 8, CA CTRLs *n* = 10, and TB cases EOT *n* = 20.

The levels of 6 analytes (Granzyme‐A, Granzyme‐B, Perforin, sFasL, IL5, and GM‐CSF) were measured in the BAL fluid and plasma of TB patients at diagnosis and at the end of treatment (EOT), as well as controls using MAGPix technology. When comparing the analyte profiles measured at diagnosis and EOT in the BAL fluid of TB participants, we found that the profiles of the two different time points differ from each other (Fig. 6a). Furthermore, the analyte profiles of BAL fluid of TB participants at both time points differed from that of CA controls (Fig. 6a). When comparing the profiles in the plasma of these patients at diagnosis and EOT, no differences were seen (Fig. 6b). Statistical analysis on the analyte levels in the BAL fluid of the TB patients and CTRLs revealed, that Granzyme‐B (*P* < 0.01) and Perforin (*P* < 0.05) levels are significantly lower at EOT when compared to Diagnosis (Dx) (Fig. 7b and e). EOT levels of Granzyme‐B (*P *< 0.05), sFasL (*P *< 0.01), and Perforin (*P *< 0.05) were also significantly different from CTRLs (Fig. 7b, c, and e).

## Discussion

In the current study, we aimed to investigate whether the so‐called regulatory “killer” B‐cells are present during tuberculosis and if there was an effect of treatment on the frequency of these cells. Previous studies suggest that these cells are present during autoimmune disease and may play an essential role in the regulation of inflammation 16, 17. Furthermore, there are characteristics about the regulatory “killer” cells that lead us to think that these cells (even though low in frequency) may play a role during tuberculosis as well, such as through the induction of these cells by binding of *M. tuberculosis* CpG motifs to TLR9 10.

The phenotype of B‐cells was assessed by means of flow cytometry to identify cells expressing FASL and IL5RA, which are markers associated with and used to identify killer B‐cells 9. Even though there were no significant differences, apart from the difference in the frequency of IgM^−^ cells expressing IL5RA, the data suggest that there is a trend to higher expression of FASL and IL5RA by cells from CTRLs compared to TB cases. Interestingly, there is an appearance of IgM^−^ cells expressing both FASL and IL5Ra at month 1 of treatment, which increases by M6. The expression of FASL and IL5RA in IgM^+^ cells also increases during treatment, but appears earlier (week 2) during treatment. These results indicate the treatment may be inducing an increase in the expression of FASL by B‐cells and that higher expression is associated with reversion back to healthy (or latently infected state) status, as the data show that B‐cells from healthy controls has a higher expression of FASL compared to TB cases at diagnosis.

To assess the level of B‐cell activation and immune exhaustion, we evaluated frequency of B‐cells expressing CD40 and PD1. IgM^+^ B‐cells expressing CD40 increased over time, whereas IgM^−^ B‐cells expressing CD40 only started increasing by month 1 of treatment, with lower frequencies compared to IgM^+^ B‐cells. This suggests that there is an increase in the activation of B‐cells during treatment, which implies that treatment may be inducing activation of B‐cells. Cliff and coworkers showed that B‐cell associated activity increases as successful treatment progresses 18.

To assess the effects of anti‐TB treatment on the expression of FASL by B‐cells at the gene level, we evaluated the changes in transcript levels over time. The results indicated that there is an increase in the levels of both genes by month 6 of treatment. In a previous study, we found that the levels of *FasL* and *Il5Ra* of the untreated TB cases were lower when we compared it to that of community controls (data not shown) 19. These results correlate with the phenotypic results which indicates that there is an increase in the surface expression of FASL and IL5RA during treatment, even though those changes are not statistically significant.

Analyte levels at the site of disease (BAL fluid) was evaluated by means of the multi‐cytokine platform. sFasL secretion increased by the end of treatment, where perforin and granzyme‐B levels were reduced at the end of treatment. The increase in sFasL in BAL fluid by the end of treatment also correlates with the phenotypic and gene expression results. FasL expression is mostly attributed to CD8+ T‐cells and NK cells in response to viral infections 20, 21. Although B‐cell frequencies in the lung is low, there is increased infiltration of B‐cells into the lung during inflammation 22, 23, 24, 25. Thus, B‐cells may also be contributing to the sFasL levels in the lung.

Collectively, these results show that TB treatment induces an increase in the expression of FASL by B‐cells. These results are in agreement with studies which show that there is an increase in the expression of FASL by B‐cells during parasitic infection and autoimmune disease 8, 26. Lundy and coworkers (2001) show that B‐cells from mice infected with *Schistosoma mansoni* had a higher expression of FASL compared to healthy mice 8. Similarly, another study shows an increase in B‐cell FASL expression during airway inflammation and asthma, as well as systemic lupus erythematosus (SLE) 26, 27, 28. Increased FASL expression by B‐cells may have different effects, depending on the disease or infection. Culture experiments and killing assays carried out by Klinker and coworkers showed that B‐cells stimulated with CD40L and IL‐5‐induced FasL‐dependent apoptosis of CD4+ T‐cells, and that these B‐cells target T‐cells based on the specificity of the antigen 9. Thus, increased FASL expression may have a protective role in the case of autoimmune disease, where apoptosis of CD4^+^ T‐cells leads to decreased inflammation. In contrast, apoptosis of Th1 cells may cause a skewed balance between Th1 and Th2 cells, leading to airway inflammation and asthma due to cytokines produced by Th2 cells.

Here, the increase in the expression of FASL during treatment implies that these regulatory “killer” B‐cells may play a protective role during TB. One hypothesis may be that these regulatory B‐cells are somehow inducing activation of T‐cells, which later undergoes apoptosis 29, 30, 31. An alternative hypothesis may be that these regulatory B‐cells are inducing apoptosis of T‐cells via FASL, as a means to rid the host of the bacteria. This process is well documented for myeloid cells 32. B‐cells are infected by *M. tuberculosis* through a process of micropinocytosis 33, 34 and express death receptors. The manner in which a general loss of T‐cells occur during tuberculosis is not well defined 35. Literature states that FasL plays a role during activation‐induced cell death 29, 36, 37, 38. When T‐cells are activated, there is an increased expression of Fas by these cells and they become sensitized to FasL‐induced apoptosis 37.

Even though the data in the relatively small study points in the same direction as the studies above, we acknowledge that the observations are preliminary and that no definite conclusions can be drawn from our findings. The studies need to be replicated and expanded by both our research group and that of others. This will then contribute further to our understanding of the phenotype of these B‐cells induced upon completion of anti‐TB treatment. However, functional studies are required to further elucidate the role of the “killer” B‐cells during TB.

## Author Contributions

ICvR holds an MRC bursary for MSc studies. GW holds the DST/NRF SARChI Chair for TB Biomarkers Research.

### Conflict of interest

The authors declare no commercial or financial conflict of interest

## Supporting information

Additional supporting information may be found in the online version of this article at the publisher's web‐site.

**Table S1**. Cohort for B‐cell phenotypic analysis.
**Table S2**. Cohort for Gene expression analysis.
**Table S3**. Cohort for Luminex.
