# Supplementary material for: B‐cells with a FasL expressing regulatory phenotype are induced following successful anti‐tuberculosis treatment
Source: Immun Inflamm Dis. 2016 Dec 27;5(1):57–67. doi: 10.1002/iid3.140 (PMC5322165; doi:10.1002/iid3.140)
Supplement: Supplementary file 1 — Table S1. Cohort for B‐cell phenotypic analysis. Table S2. Cohort for Gene expression analysis. Table S3. Cohort for Luminex. [file IID3-5-57-s001.doc]

**Supplementary data**

**Table 1: Cohort for B-cell phenotypic analysis**

|  | **TB** | **CTRL** |
| --- | --- | --- |
| **No. of Females** | 2 | 10 |
| **No. of Males** | 11 | 5 |
| **Median Age (years old)** | 50 | 38 |
| **Gene Xpert-MTB/RIF Assay** | POSITIVE | NEGATIVE |
| **Quantiferon status** | NA | POSITIVE |
| **Sputum-culture status (Dx)** | 3 NEGATIVE | NA |
|  | 10 POSITIVE |  |

Table 2: Cohort for Gene expression analysis

|  | **TB** |
| --- | --- |
| **No. of Females** | 8 |
| **No. of Males** | 11 |
| **Median Age (years old)** | 28 |
| **QuantiFERON status (Dx)** | NA |
|
| **Sputum-culture status (Dx)** | POSITIVE |

Table 3: Cohort for Luminex

|  | **TB (Dx)** | **CA** | **TB (M6)** |
| --- | --- | --- | --- |
| **No. of Females** | 4 | 7 | 9 |
| **No. of Males** | 4 | 3 | 11 |
| **Median Age** | 41 | 60 | 31 |
| **Gene Xpert/Sputum-culture** | POSITIVE | NEGATIVE | NEGATIVE |
